# Acceptability and Feasibility of a Prototype Regional Disaster Teleconsultation System for COVID-19 Pandemic Response: Pilot Field Test

**DOI:** 10.2196/73078

**Published:** 2025-08-19

**Authors:** Stephanie Ludy, Mari-Lynn Drainoni, Lauren Schmidt, Mark Litvak, Julianne Dugas, Eric Goralnick, Paul D Biddinger, Tehnaz Parakh Boyle

**Affiliations:** 1 Department of Emergency Medicine, Harvard Medical School, Brigham and Women’s Hospital, Boston, MA, United States; 2 Department of Emergency Medicine, Harvard Medical School, Massachusetts General Hospital, Boston, MA, United States; 3 Department of Medicine, Section of Infectious Diseases, Boston University Chobanian and Avedisian School of Medicine, Boston Medical Center, Boston, MA, United States; 4 Department of Health Law, Policy and Management, Boston University School of Public Health, Boston, MA, United States; 5 Evans Center for Implementation and Improvement Sciences, Boston University Chobanian and Avedesian School of Medicine, 801 Albany Street, Boston, MA, United States; 6 Department of Pediatrics, Boston Medical Center, Boston, MA, United States; 7 Department of Pediatrics, Boston University Chobanian and Avedisian School of Medicine, Boston Medical Center, 801 Albany Street, Rm 4027, Boston, MA, 02119, United States, 1 617-414-3682, 1 617-414-4393; 8 Department of Emergency Medicine, Boston Medical Center, Boston, MA, United States; 9 Region 1 Disaster Health Response System, Boston, MA, United States

**Keywords:** disaster medicine, teleconsultation, regional medical programs, COVID-19, tele-critical care

## Abstract

**Background:**

Disaster telehealth can be used to provide rapid access to remote specialty expertise and virtual surge capacity for overwhelmed local clinicians. The Regional Disaster Health Response System (RDHRS) is developing a disaster teleconsultation system for cross-jurisdictional care in the United States. In 2020, the Region 1 RDHRS provided Massachusetts hospitals access to disaster teleconsultation services with out-of-state critical care experts during the first wave of the COVID-19 pandemic response.

**Objective:**

We aimed to field-test (1) the acceptability and feasibility of using a prototype, web-based disaster teleconsultation platform with minimal-to-no user training and (2) the feasibility of deploying a national volunteer expert pool to access out-of-state expertise.

**Methods:**

This was a prospective, mixed methods, observational study. We recruited field clinicians from Massachusetts hospitals and out-of-state critical-care physicians as experts for a 2-week pilot (June 2020). Experts were trained to use a prototype platform, while field clinicians received a just-in-time tool. Field clinicians requested teleconsultations for hospitalized patients with COVID-19 (clinical call) or simulated patients (test call). We collected demographics, call performance data, and Telehealth Usability Questionnaire (TUQ) ratings to measure acceptability (primary outcome; total usability score ≥6 of 7) and feasibility (secondary outcome; interface, interaction quality, and reliability items), and interviewed participants. We report descriptive statistics and key themes using the Technology Acceptance Model framework.

**Results:**

Ten experts from 6 states and 17 field clinicians from 4 hospitals participated. All experts and 10 field clinicians completed postpilot questionnaires (74% response overall). Of these, 20% had previously used telemedicine in a disaster. In total, 50 test calls and no clinical calls were logged. Most (70%) made ≥1 call; 22% (95% CI 10%‐34%) connected successfully. The median time to connect was 1.6 (IQR 3.2) minutes. Among field clinician respondents, 50% used smartphone devices, 40% hospital desktop computers, and 10% laptop computers to access RDHRS teleconsultation services. Calls failed due to platform routing errors (49%), hospital computers without cameras or microphones (10%), firewalls (8%), and expert notification failures (5%). The mean total usability score was 5.6 (SD 1.3). TUQ item scores were highest in usefulness (mean 6.0, SD 1.1) and ease-of-use (mean 6.0, SD 1.4), and lowest in reliability (mean 2.4, SD 1.4). Participants were comfortable using the platform. Those with difficulty identified discomfort with technology as the cause. All experts were willing to participate in a national expert registry and obtain emergency licensure, and most (80%) were willing to serve on a volunteer, unpaid basis.

**Conclusions:**

Clinicians found the prototype platform acceptable, but the workflow requires revision to reduce call failure and improve feasibility and reliability for future use with minimal-to-no training. Using familiar clinical workflows for emergency consultation and mobile devices with camera and microphone capabilities could improve call performance and reliability.

## Introduction

In 2018, the Administration for Strategic Preparedness and Response launched the Regional Disaster Health Response System (RDHRS) program as part of the national strategic response to disasters in the United States [1]. The goal of an RDHRS is to establish multi-state partnerships and enhance local health care assets to support care delivery when catastrophic events overwhelm local and state capacity and capability. Telehealth is an important RDHRS tool to expand access to specialty care and increase surge capacity. Disaster telehealth services during hurricanes, wildfires, and pandemics have helped maintain access to health care when traditional in-person services are disrupted [2-5]. Delivering telehealth services across state lines and jurisdictions has been limited by poor interoperability of telehealth platforms, variable infrastructure, and regulatory barriers related to licensure, credentialing, and reimbursement [6].

The Region 1 RDHRS, which serves the 6 New England states, is developing a disaster teleconsultation system to provide rapid, temporary, cross-jurisdictional access to clinical specialists to support disaster medical response. We previously demonstrated proof-of-concept for a model teleconsultation system operating within a regional medical emergency operations center (MEOC) in simulated disasters [7]. In this model system, the regional MEOC coordinates access to an expert clinician pool to provide temporary disaster teleconsultation services to field clinicians in affected hospitals. While this model had high clinician acceptance, it required a simpler, more intuitive communication interface to support efficient response in real-world settings. The initial prototype model used a telehealth platform in clinical use at one academic medical center to connect to hospitals outside its health system. It required a trained telehealth coordinator to triage patients by telephone, connect bedside clinicians with remote expert teleconsultants, and guide just-in-time installation and use of software. However, dependence on external guidance for technology use and manual triage hampered workflow efficiency and system throughput.

In February 2020, as the first COVID-19 cases skyrocketed, 3 factors motivated the Region 1 RDHRS team to develop and field-test another prototype platform for real-world use. First, large surges in critical patients produced severe equipment and workforce shortages, overwhelming regional critical care capacity [8]. Second, COVID-19 led to the widespread adoption of video technology that made patients, providers, and health care organizations more receptive to using telehealth tools [9]. Third, the Coronavirus Aid, Relief, and Economic Security Act and state emergency laws and waivers lifted legal, regulatory, and reimbursement barriers to the implementation of regional telehealth systems [10,11].

In early March 2020, we collaborated with industry partners to configure a new disaster telehealth platform for pilot field implementation. Our primary objective was to test the feasibility and acceptability of providing Massachusetts hospitals temporary access to disaster teleconsultation services with out-of-state critical care experts during the first wave of the COVID-19 pandemic response. Our secondary objectives were to test the feasibility of deploying a national volunteer expert pool and identify best practices and policy recommendations to support implementation in future disasters.

## Methods

### Study Design

This was a prospective observational study. We used mixed methods to capture clinician ratings and feedback after a 2-week pilot field implementation of the prototype RDHRS teleconsultation system. We included a test call strategy to evaluate call performance in conditions approximating real-world use if clinical use lagged. We followed STROBE (Strengthening the Reporting of Observational Studies in Epidemiology) guidelines for reporting observational studies.

### Ethical Considerations

The Boston Medical Center/Boston University Medical Campus institutional review board approved this study and granted an exemption under Category 2 of the Human Research Protection Program policies and procedures. Although we used an informed consent process, the study qualified for a *Waiver of Documentation of Informed Consent* due to the minimal risk of harm to participants and the nature of the study procedures, which typically do not require written consent outside of a research context. Participant data was deidentified and assigned a unique study identifier, with the master code stored separately and accessible only to the study team. Participation was voluntary and unremunerated.

### Setting

The study was conducted in Massachusetts during the first wave of COVID-19 response in 2020. We gave Massachusetts hospitals temporary access to disaster teleconsultations using a national volunteer pool of critical care experts. Based on participating hospital needs, disaster teleconsultation services were available on demand for 12 hours/day (5 PM-5 AM) from June 1 to June 15, 2020.

### Eligibility and Recruitment

We recruited subject matter experts (SMEs) from the Society for Critical Care Medicine as RDHRS teleconsultants via email invitation to a list of 100 member volunteers interested in testing new technologies for remote care delivery provided by the Society. Eligible SMEs had a full medical license in good standing in any US state, territory, or district. Participation was part-time.

All Massachusetts hospitals were invited to participate as field test sites by email invitation sent by the Massachusetts Hospital Association and local health care coalitions on behalf of the RDHRS. Hospitals interested in accessing RDHRS teleconsultation services were introduced to the goals of the pilot in follow-up virtual meetings with the RDHRS team. There was no fee for service use. SME and hospital participation was voluntary.

### Prototype RDHRS Teleconsultation Platform and Workflow

After testing off-the-shelf video-conferencing platforms, we selected a Health Insurance Portability and Accountability Act (HIPAA)–compliant telehealth platform (Bluestream Health, Inc.) for disaster-relevant capabilities. This commercially available, web-based application had low bandwidth requirements, did not require software or hardware installation, and was device-agnostic and interoperable to support a distributed care delivery model. The platform was configured for relevant workflows (virtual interpreter and tele-emergency services) with remote high-throughput triage capability, virtual team functionality, and rapid scalability, and it was FirstNet-certified for priority network access. Key security features included end-to-end encryption for all data transmissions, secure communication channels to protect patient information during teleconsultations, robust authentication protocols to verify user identities, and restricted access to safeguard sensitive health data.

### Pilot Field Implementation Process

#### SME Onboarding

SMEs were onboarded as RDHRS teleconsultants as follows: (1) each out-of-state volunteer obtained an emergency temporary medical license to comply with Massachusetts law, (2) physicians unable to use their existing malpractice insurance signed a volunteer physician practice agreement with an RDHRS partner institution (Boston Medical Center) to receive secondary liability coverage during pilot field implementation, and (3) all SMEs tested device and internet connections and were trained to technical competency in platform use. SMEs also received a just-in-time user manual with graphic instructions on how to receive and answer video consultations for reference during and after training.

#### Hospital Onboarding

Each participating hospital received an implementation toolkit describing (1) RDHRS program goals, (2) existing emergency legislation and regulatory waivers, (3) SME licensure and credentials, (4) minimum technology requirements, and (5) teleconsultation service parameters. Hospitals used emergency credentialing procedures written in their bylaws. Hospital technology teams were given information to bypass firewall protections by whitelisting.

#### User Registration

To maintain cybersecurity standards, each SME and hospital user was registered by the technology vendor prior to service activation. This included configuring the platform to route consult requests from registered hospitals through the MEOC to the SME.

Field clinicians were given password-protected access and electronic copies of the just-in-time user manual but otherwise received no training or exposure to the platform. Field clinicians were instructed to request teleconsultation for hospitalized adult patients with COVID-19 (clinical call) or simulated patients (test call) on demand.

### Assessment Tools

#### Quantitative Tools

After the pilot period, all participants completed data forms to report demographics and rate the platform on the Telehealth Usability Questionnaire (TUQ). The TUQ has 21 items validated to assess telehealth platform usefulness, ease of use, interface quality, interaction quality, reliability, and satisfaction on 7-point Likert scales [12]. A total usability score (TUS) can be calculated as the mean user rating for all 21 items (maximum score=7).

Field clinicians also received a brief questionnaire to query user comfort using the RDHRS prototype system without prior training or exposure, device types used to request teleconsultations, success completing a video call on the first attempt, reasons for call failure, success completing a video call during any attempt, comfort making future teleconsultation requests, user preferences for device type to receive video consults during disasters, willingness to use personal devices, and preferences for notification of teleconsultant availability.

#### Qualitative Tools

To supplement survey assessments and contextualize our findings, we also conducted individual interviews with those who completed surveys. We designed a semistructured interview guide to explore participants’ overall perception and comfort level with the platform, device preferences, challenges with use, and recommendations for future implementation.

### Outcomes

The primary outcome, acceptability, was measured as TUS≥6 of 7. The secondary outcome, feasibility, was measured via select TUQ items in interaction quality and reliability domains.

Call performance measures were recorded and downloaded from the platform, including the frequency of calls attempted, calls connected, video calls completed successfully, reasons for call failure, and wait times. A connection was successful when a field clinician completed a synchronous video call with the SME. Reasons for call failure included user cancellation (call canceled manually by the requester), orphaned call (call canceled by the server when the SME could not be contacted or the server lost contact with the requester before the SME answered), call routing error (error in call routing from the hospital to the MEOC), expert notification error, device problem, and connection problem.

### Data Collection

Questionnaires were administered electronically immediately after the pilot period ended and stored using the REDCap (Research Electronic Data Capture; Vanderbilt University) platform hosted by Boston University [13]. All participants were given 2 weeks with 3 email reminders to complete questionnaires.

After survey completion, brief interviews (30 min) with willing participants were audio-recorded using Zoom Pro by 2 study investigators. Audio recordings were transcribed verbatim by a third-party transcription service.

### Sample Size

As a pilot field test, this study was not powered to detect statistically significant outcomes. We determined that a minimum of 30 experts would be required to ensure 24/7 on-demand teleconsultation. Our staffing model assumed that each physician would volunteer for three 4-hour shifts during the 2-week pilot period. Based on preliminary data, we needed approximately 20 participants to estimate acceptability with adequate precision. Assuming each encounter involved 1 field clinician and 1 expert, we anticipated a minimum of 10 teleconsultation encounters during the pilot period.

### Analysis

#### Quantitative Analysis

We used SAS (version 9.4; SAS Institute) software for descriptive statistics and NVivo (version 12; Lumivero) software for qualitative coding and analysis. We report means with SD, medians with IQR, and proportions with 95% CI as appropriate.

#### Qualitative Analysis

To code transcripts, we revised an inductive codebook developed previously [7]. Two investigators (SL and LS) independently reviewed and coded 2 transcripts using the prior codebook and met to establish consensus and revise codes for the final codebook. The 2 investigators then independently coded each transcript using the final codebook, with frequent meetings to reconcile discrepancies by consensus. After coding was complete, themes were created and mapped to the Technology Acceptance Model framework to identify factors influencing acceptance of disaster teleconsultation services by SMEs and field clinicians [14]. In this theoretical framework, how providers perceive the *usefulness* (use enhances or diminishes care delivery) and *ease-of-use* (use is easy or difficult) of a technology solution affects their *attitude* (positive or negative emotion) and *behavioral intention* (willingness) to adopt and use it in the future.

## Results

### User Statistics

Of 100 Society of Critical Care Medicine members who volunteered to test new technologies, 66 SMEs were eligible for recruitment based on board certification. Among these, 27/66 (41%) responded to an invitation to volunteer as an RDHRS teleconsultant. Ultimately, 10/66 (15%) eligible SMEs from 6 US states completed the onboarding process and participated in the study.

In total, 4 community hospitals and 3 tertiary care centers in Massachusetts expressed interest in participating in the pilot. Of these, 2 community hospitals and 2 tertiary care centers completed their administrative review and onboarding in time to participate in the pilot. A total of 17 board-certified internal and emergency physicians from these 4 hospitals participated as field clinicians.

Of these, 10 field clinicians and 10 SMEs completed post-pilot questionnaires (75% response rate). Table 1 summarizes participant demographics.

**Table 1.** Demographic data for participating field clinicians (n=10) and expert teleconsultants (n=10), including self-reported sex, age, specialty, years of clinical experience, primary practice setting, and prior utilization of telemedicine either clinically or in a prior disaster setting.

Demographics	Values
Sex, n (%)	
Male	11 (55)
Female	9 (45)
Age in years, median (IQR)	44 (14)
Medical specialty, n (%)	
Anesthesiology critical care	4 (20)
Emergency medicine	7 (35)
Internal medicine/hospitalist	1 (5)
Internal medicine/critical care medicine	3 (15)
Pulmonary/critical care medicine	4 (20)
Surgical critical care	2 (10)
Other	0 (0)
Years of clinical experience, n (%)	
<5 years	0 (0)
5‐9 years	6 (30)
10‐19 years	7 (35)
≥20 years	7 (35)
Primary practice setting, n (%)	
Academic tertiary care hospital	9 (45)
Community hospital	10 (50)
Other	1 (5)
Best estimate of how many times you have previously used telemedicine to care for or consult on a patient in any setting, n (%)	
Never	8 (40)
<5 times	2 (10)
6‐10 times	1 (5)
>10 times	9 (45)
Best estimate of how many times you have previously used telemedicine to care for or consult on a patient in a disaster setting, n (%)	
Never	16 (80)
<5 times	0 (0)
6‐10 times	2 (10)
>10 times	2 (10)

### Evaluation Outcomes

In total, 50 test calls and no clinical calls were logged. While only 3 of the 10 field clinician respondents (30%) reported success on their first attempt, most (70%) successfully completed at least 1 video call during the pilot period. Among the 50 test calls, only 22% (95% CI 10%‐34%) connected successfully (Table 2). The median time to connect to an expert teleconsultant was 1.6 (IQR 3.2) minutes.

**Table 2.** Call frequency and performance across all field sites (Hospital 1, Hospital 2, Hospital 3, and Hospital 4) for all attempted calls (n=50) and the subset of calls routed through the virtual Region 1 RDHRS^a^ MEOC^b^ (n=23).

	Total calls attempted	Calls routed through virtual MEOC
Call frequency by site, n (%)		
Hospital 1	28 (56)	5 (22)
Hospital 2	12 (24)	8 (35)
Hospital 3	5 (10)	5 (22)
Hospital 4	5 (10)	5 (22)
Patient registered, n (%)	50 (100)	23 (100)
Consult request initiated, n (%)	50 (100)	23 (100)
Call routed to RDHRS, n (%)	23 (46)	23 (100)
Expert notified, n (%)	23 (46)	21 (91)
Expert responded, n (%)	21 (42)	19 (83)
Connection established, n (%)	15 (30)	15 (65)
Video call successful, n (%)	11 (22)	11 (48)
Video problem during call, n (%)	5 (10)	5 (22)
Audio problem during call, n (%)	7 (14)	7 (26)

a RDHRS: Regional Disaster Health Response System.

b MEOC: medical emergency operations center.

Table 3 details the reasons for call failure (n=39) recorded by the Region 1 RDHRS platform. Half (49%) of the failed calls were due to a call routing error in Hospital 1, and 14 (36%) failed for >1 reason. Among 23 test calls routed correctly to the MEOC, 48% (95% CI 27%‐69%) were successful video call connections (Table 2).

**Table 3.** Reasons for call failure (N=39) recorded by the platform. Counts exceed 100% as calls can fail for multiple reasons.

Reasons for call failure	Reasons, n (%)
User canceled call	12 (31)
Orphaned call	8 (21)
Call routing error	19 (49)
Expert notification error	2 (5)
No connection logged	4 (10)
Device problem	4 (10)
Connection problem	3 (8)

Among field clinician respondents (n=10), 50% used smartphone devices, 40% used hospital desktop computers, and 10% used a laptop computer to access RDHRS teleconsultation services. All hospital desktop computer users failed to connect their first call. Lack of camera or microphone capability (30%) and lack of video (40%) or audio (20%) during the call were common reasons for call failure reported by field clinicians. Fewer reported difficulties logging in to the platform (20%), adding a patient (10%), or initiating a video call (20%).

The mean TUS was 5.6 (SD 1.3; Table 4). TUQ item scores were highest in usefulness (mean 6.0; SD 1.1) and ease of use (mean 6.0, SD 1.4) and lowest in reliability (mean 2.4, SD 1.4).

**Table 4.** Telehealth Usability Questionnaire (TUQ) item mean scores with SD and median scores with IQR. Participants (N=20) rated agreement with each item on a 7-point Likert scale (1=disagree, 7=agree). The total usability score (TUS) is the average for all items (maximum score=7).

Domain and item	Participants, n	Mean (SD)	Median	Quartile	
				Lower	Upper
Usefulness					
Telemedicine improves my ability to provide or receive remote medical consultation during a disaster.	20	6.0 (1.1)	6.0	5.0	7.0
Telemedicine saves me time when communicating with another provider.	16	5.0 (1.8)	5.5	4.0	6.5
Telemedicine provides for my needs as a health care provider.	16	5.3 (1.4)	5.5	4.0	6.5
Ease of use and learnability					
It is simple to use this system.	18	5.5 (1.5)	6.0	5.0	7.0
It is easy to learn to use the system.	18	6.0 (1.4)	6.5	6.0	7.0
I believe I could become productive quickly using this telemedicine system.	18	5.8 (1.3)	6.0	5.0	7.0
Interface quality					
The way I interact with this system is agreeable.	16	5.6 (1.3)	6.0	5.0	6.5
I like using this telemedicine system.	17	5.3 (1.4)	5.0	5.0	6.0
The telemedicine system is simple and easy to understand.	19	5.7 (1.6)	6.0	4.0	7.0
This telemedicine system is able to do everything I would want it to be able to do.	15	4.4 (2.1)	5.0	2.0	6.0
Interaction quality					
I can easily talk to the clinician using this telemedicine system.	15	5.4 (1.6)	6.0	5.0	7.0
I can hear the clinician clearly using this telemedicine system.	15	5.5 (1.6)	6.0	5.0	7.0
Using this telemedicine system, I am able to express myself effectively.	16	5.8 (1.6)	6.0	5.5	7.0
Using this telemedicine system, I can see the clinician as well as if we met in person.	15	5.3 (1.6)	6.0	4.0	6.0
Reliability					
I think the consultations I give or receive over this telemedicine system are the same as hospital-based consultations.	17	4.2 (1.8)	4.0	3.0	6.0
Whenever I make a mistake using this system, I can recover easily and quickly.	11	4.2 (2.1)	4.0	2.0	6.0
This telemedicine system gives error messages that clearly told me how to fix problems.	19	2.4 (1.4)	2.0	1.0	3.0
Satisfaction and future use					
I feel comfortable communicating with the clinician using this telemedicine system.	15	5.6 (1.6)	6.0	5.0	7.0
This telemedicine system is an acceptable way to give or receive online consultation.	16	5.9 (1.5)	6.0	6.0	7.0
I would use this disaster telemedicine service again.	16	6.0 (1.5)	6.0	6.0	7.0
Overall, I am satisfied with this telemedicine system.	16	5.4 (1.8)	6.0	4.5	7.0
Total usability score	20	5.6 (1.3)	5.9	4.9	6.5

All participants felt comfortable using the RDHRS platform to access disaster teleconsultation services. Most (80%) field clinicians preferred to use personal or institutional smartphones or tablet devices. All SMEs were willing to participate in a national expert registry, including on short notice. All were willing to obtain out-of-state emergency licensure, and 8 in 10 were willing to serve on a volunteer, unpaid basis. Almost all (90%) SMEs were willing to provide teleconsultation services from a personal device.

In total, 6 field clinicians and 6 SMEs completed interviews. Table 5 summarizes key themes with representative quotes. Most physicians perceived the platform as simple and easy to learn, and those who had difficulty learning the system identified discomfort with technology as the primary cause. When video was limited, providers could still communicate via an audio call or real-time chat function. Most field clinicians agreed that simplifying the 2-step process of connecting to SMEs would improve system workflow. Almost all SMEs desired more platform training to support real-time technical assistance for field clinicians during the call.

**Table 5.** Key themes and representative quotes from physicians regarding perceived usefulness and ease-of-use, attitudes, behavioral intention to use, and future recommendations for using the prototype model system for disaster teleconsultation services.

Theme	Representative quotes from physicians
Usefulness	
Access to teleconsultants can enhance bedside physician capabilities	*“*I think it’s especially useful when there are novel issues for which providers are not familiar with.” *“*I think having that ability to have this type of a resource would be invaluable in that situation.*”* *“*I would clearly just use it for um, for uh, for a disease or a skill that I don’t have.” *“*Sort of the value proposition here is completely access to a bunch of specialty services that you don’t have at a small hospital or wherever that I will be in the middle of nowhere. Um, just that safety net feels great.”
I thef workflow is not efficien,t it may be underused	*“*…the login for sure. I mean, especially in a disaster setting, I think people really have a very little patience to try to figure out how to get into the system in a short amount of time” “The greater, the disaster situation, the sort of greater time crunch and acuity and volume, you have, you may feel less inclined to, um, to stop it, utilize this”
Internal Context	
Individual provider comfort level with telemedicine may limit usability	*“*For me everything technical is a challenge.*”* *“*Not everyone is as familiar with smartphones or applications or even computers*”* *“*I think it’s gonna also come down to like my, my degree of comfort or familiarity with like what it is that I’m treating in the first place.*”* *“*I’ll say that a lot of our older attendings who probably will be using this to when a disaster, right, um, are less comfortable with it.*”*
Access to proper devices will be essential	*“*I had no audio capabilities on my computer at the hospital and my phone wasn’t working.” *“*I use my desktop, which does not have a microphone or video capabilities. I was able to log in, um, which I could from my phone for some reason.*”*
Site-specific limitations mus bet addressed in advance	*“*I think that I had roadblocks within the hospital for various reasons.*”* *“*…it’s a running joke here about our WiFi*”* *“*There were a large number of your calls that were going to, that were routing to the wrong call center, essentially.*”* *“*I imagine it was the firewall that they gave me trouble with, um, the audio and video on the first call when I was at MGH. The one that I did that was successful was at home.*”*
Ease of use	
System was simpl;e howeve,r logging in to the system proved difficult in practice	*“*It was pretty self-explanatory to me once I got into the system.*”* *“*…there weren’t, you know, there weren’t too many options or buttons, which is a good thing*”* “You know, from the initial training, it seems like it should have gone very easily and it didn’t seem to be quite as intuitive” “The login password would be the most difficult part to manage.” “The technical parts of trying to log in and having separate passwords and having a different user login, anything to simplify that would be amazing.” “Whatever that extra button is for the request to make it, to alert me. Uh, I found that challenging”
The interface quality was strong but needs improvements	“The quality of the video and the audio is really good even on my phone.” “Overall the interface is pretty user friendly with the very few buttons and the buttons on the side were pretty self-explanatory” “…trying to have a conversation when every three or four seconds the screens are freezing, you’re just going to say, ‘Uh, I don’t have time for this.” “I had challenges with the video function again, not knowing um, exactly what, uh, how to activate it from the other end.”
Workflow improvements will assist just-in-time training for future disasters	“I did have to read through the emails and the sheets very carefully to make sure I was doing everything right. Um, so that was a little bit of a barrier.” *“*…would take some practice ahead of time because again, I mean, for me to just jump in and start doing screen-sharing, I’m not sure how easy that would be.” “…because it’s all web based, it was seamless and, you know, very easy from that regard.” “If I didn’t have the PDF, I’m not sure. I haven’t tried it without following the instructions you sent”
Attitudes and behavioral intention to use	
Institutional support and liability protections could incentivize use	“…as long as the institutional support and, and liability reasons are there, you know, supported, then I think it’d be really easy to use”
Access and awareness of system availability could influence use	“There’s nothing that would make me not want to use the system, other than the inability to access it or forgetting that I had it.”
Recommendations for future use	
Workflow improvements to improve simplicity	“Something as simple as the password reset” “There’s that one extra step to make it an active consult.” “…whatever that extra button is for the request to make it, to alert me. Uh, I found that challenging” “There’s the completed consult and then request a consult and then like active consult. Um, it was sitting in sort of that holding middle.” “…so the less steps the better, or at least the more guided steps, the better as well, too.”
Expert teleconsultant’s role needs clarification	“Not having a clear line of communication in terms of what’s being expected of the consultation.” “So potentially just more expertise on the side of, of us. Um, as the, as the supposed experts on implementing the system.”
Institutional support and “super users” at each site could be invaluable	“The challenge in getting something like this up in, you know, a couple of days without super users at the various sites.” *“*I think overall institutional support, societal support, medical, legal, liability…” “Eventually the legal or public with legal ramifications in public view.” “…probably honestly a champion in every institution who can bring this about and to let the not only their staff know, but make sure it’s a discussed in the faculty meetings”

Overall, the biggest barrier to successful connections was issues related to the lack of audiovisual capabilities on hospital computers, access to reliable and consistent mobile or wireless internet connections in clinical workspaces, and circumventing institutional firewall restrictions. Despite technical limitations, most expressed positive attitudes and future intent to use the platform. Recommendations for future use included clarifying the role and expectations of teleconsultants, streamlining the workflow for field clinicians to minimize risk of call failures, and improving the SME’s knowledge of the system to facilitate real-time communication with field clinicians.

## Discussion

### Principal Results

This was a field test of the acceptability and feasibility of implementing a prototype RDHRS disaster teleconsultation system to deliver tele-critical care services to Massachusetts hospitals during the first wave of the COVID-19 pandemic. Clinicians found the prototype platform acceptable, but the workflow requires revision to reduce call failure and improve feasibility and reliability for future use with minimal-to-no training. We expanded access across hospital systems in real-world conditions and deployed SMEs from a national volunteer pool using emergency waivers and state emergency licensure processes, although this delayed implementation and impacted clinical use.

We were able to improve our initial prototype model in important ways [7]. We avoided software or hardware needs by deploying a web-based application. Using typical emergency department workflows and automating triage and expert notification processes that were previously done manually improved call center efficiency by reducing call wait times. However, these metrics are based on small samples under low volume conditions and did not involve patient care. Future evaluations should include broader system performance indicators, such as throughput, consult duration and quality, and user experience, ideally measured in real-world conditions.

**Figure 1.**
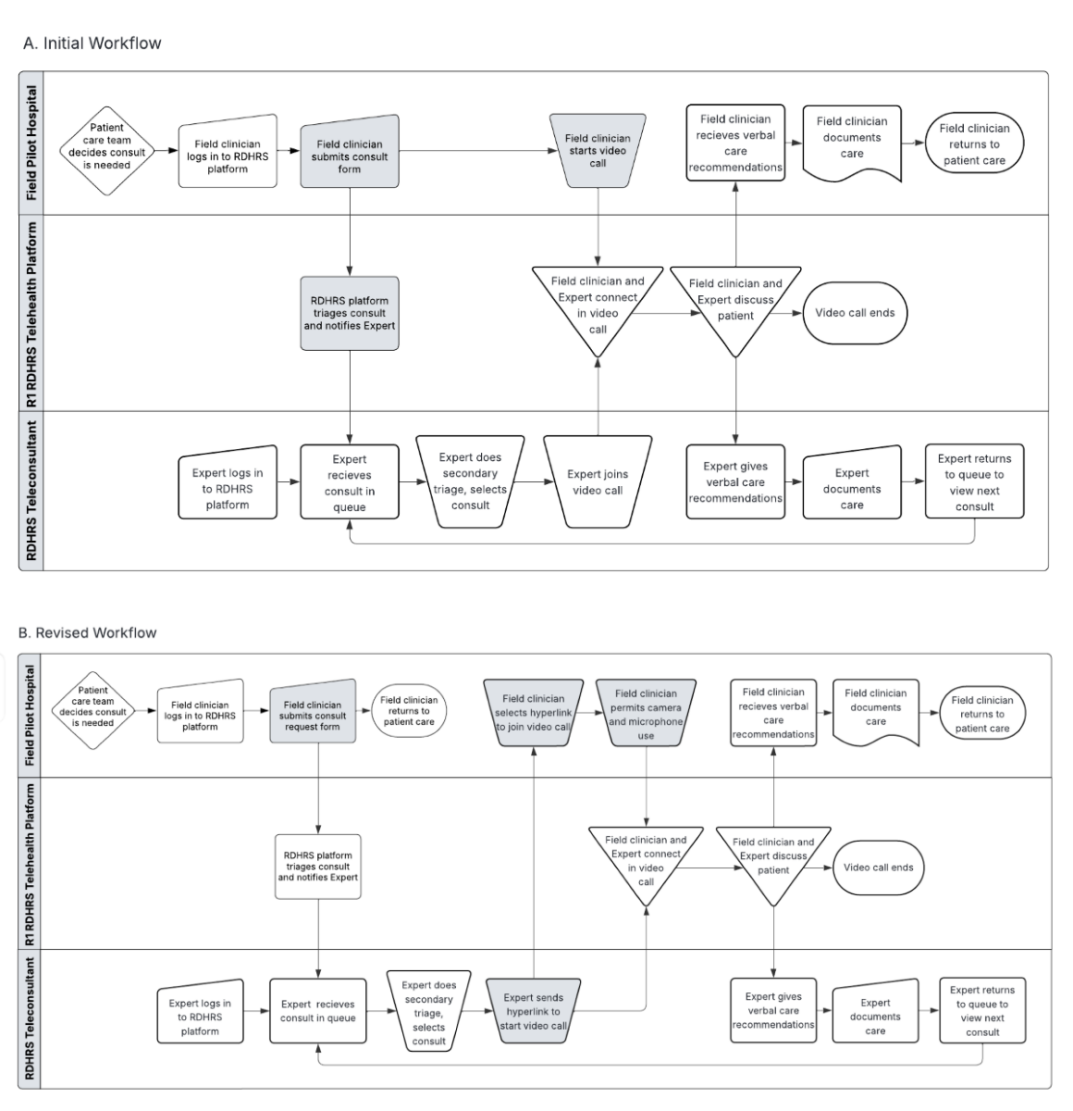
Summary of the prototype workflow. We used tele-emergency workflows to register and room patients. This involved a 2-step process where the field clinician first added one or more patients and then requested consultation. Triage and expert notification processes were automated within a virtual medical emergency operations center where consults were prioritized by illness severity and timing. The prototype R1/RDHRS (Regional Disaster Health Response System) teleconsultation system: (A) initial workflow and (B) revised workflow.

Our workflow analysis identified potential sources of call failure at platform, user, and hospital levels that require technology and system solutions to improve call center performance and reliability. Initially, overall call connection success through the MEOC was low because the technology vendor accidentally introduced a call routing error while registering one hospital. Call failures due to expert notification errors, use of hospital computers lacking cameras and microphones, and institutional firewalls were less common. Though many participants could complete a call, the major sources of call failure—orphaned and canceled calls, and call routing errors—occurred early during steps where the consult was requested and routed to the MEOC. In the initial configuration (Figure 1A), the field clinician initiated the video call connection, which required them to remain at the location of the device used to initiate the call until the SME answered. Thus, call routing errors and delayed expert response produced orphaned and canceled calls. Field clinicians also reported confusion with the 2-step process, often mistaking patient registration with consult request submission.

We have since streamlined the workflow to eliminate these errors (Figure 1B). Field clinicians (or support staff) now submit consult requests in a single step and receive confirmation. The consult request is automatically routed to the MEOC, which alerts the SME to a pending consult. The SME then initiates the video call connection by sending a text link and email invitation to the field clinician’s chosen device. The video call connection is established when the field clinician accepts either invitation. This frees the field clinician to remain active in care and may reduce orphaned or canceled calls. These changes improved call connection success in a subsequent large-scale functional exercise conducted by the RDHRS, but these data are preliminary and not yet externally validated. Future third-party assessment is needed to validate system performance.

Each hospital was onboarded individually to institutional cybersecurity standards in this pilot field test. We found this process may hinder scalability and was vulnerable to error. Registering hospitals in advance could mitigate this but requires central coordination and administrative support. Another option is to use a central web portal with an event-specific code that can be distributed via existing emergency notification systems for just-in-time access. This feature could enable an RDHRS to control system activation and deactivation centrally, make services accessible immediately but restricted to affected facilities, and scale system use to demand.

### Comparison With Prior Work

Our findings support prior studies highlighting the importance of access to suitable devices [15,16]. While the platform was device-agnostic, hospital computers without cameras or microphones accounted for many failed calls. Every participant who used a desktop computer failed their first call, and device problems constituted a third of call failures. Using designated institutional mobile devices, tablets, or personal devices could improve call reliability. A 2021 systematic review identified lack of workflow integration, outdated technology infrastructure, and user acceptance among the most commonly cited barriers for telehealth services implemented globally during COVID [15]. Postpandemic institutional technology upgrades may benefit the future implementation of disaster teleconsultation systems [17]. We recommend hospital emergency operation plans include devices with audio-visual capabilities and telecommunication network redundancy to ensure seamless access to disaster teleconsultation services.

Our experience also highlights how technology, workforce, and operational system readiness are essential to harness digital health tools for disaster response. We accelerated prototype system development and tested implementation in March 2020 as the need to expand critical care access emerged. Working with an industry partner, we configured a prototype platform for clinical use within 2 weeks. Despite federal and state emergency laws and waivers [10,11], it took an additional 6 weeks to navigate out-of-state physician licensure, hospital credentialing, and liability coverage for teleconsultants. By the time pilot implementation began in June 2020, COVID-19 cases had declined in Massachusetts, so only simulated calls were performed.

This study mirrors prior research demonstrating how variation in licensing and credentialing procedures can delay service provision, despite emergency declarations [6,18,19]. The United States lacks a national medical license. Instead, state medical boards grant physician licenses to practice medicine in that state. Hospitals and healthcare organizations use credentialing procedures to verify qualifications before permitting physicians to provide care within their institutions. The Interstate Medical Licensure Compact is 1 legal pathway to expedite licensure [20]. However, some states are not members, and full licensure is still required in each state, so the financial and administrative burden for physician volunteers would be prohibitive. Model legislation, like the Emergency Volunteer Health Practitioners Act [21], is an alternate pathway to allow license reciprocity, credentialing waivers, and liability protections during declared emergencies, but only 20 states have enacted such laws. Without adoption of national solutions, RDHRS and similar response entities will need to rely on state emergency waivers and limit scope of practice to advisory only when providing access to disaster teleconsultation services across jurisdictions.

We and others have argued that disaster telehealth uptake requires proactive rather than reactive system development [18,22]. Disasters requiring specialty care are often no-notice events, so disaster teleconsultation systems need simple, reliable, field-tested platforms and preverified, rostered clinicians to support virtual deployment within hours rather than days to weeks. A range of specialists may need to be onboarded quickly because service needs are dictated by the medical consequences of the disaster. Therefore, these systems need to be flexible to support various use cases and easy to use regardless of discipline, training, or exposure. Emulating key features of the workflow and infrastructure of established emergency call centers and providing a clear scope of practice for disaster teleconsultants could encourage adoption.

Participants referenced US Poison Centers [23] as an effective model for delivering on-demand emergency consultations. Field clinicians felt comfortable consulting Poison Center services to access medical experts by telephone. However, once the expert could view the patient via video, providers were unclear whether the interactions constituted the broader practice of medicine (telemedicine) versus offering recommendations (teleconsultation) at a distance.

While teleconsultation is a type of telemedicine encounter, there are important distinctions in provider responsibilities, licensure, liability, and patient privacy [24]. Providers must adhere to standards of in-person practice, but responsibility and liability depend on whether the teleconsultant is directly involved in care or offering advice. Many states provide a “consultation exception” to licensure, but the scope and details of these exceptions vary by state [25]. Clinicians need to verify their malpractice coverage extends to teleconsultation and includes relevant jurisdictions. They should also include the limitations of remote assessment in any medical documentation.

While HIPAA provisions were temporarily relaxed during the COVID-19 public health emergency, using off-the-shelf platforms without encryption introduces the risk of privacy breaches. Disaster telehealth platforms should use robust technical and administrative safeguards aligned with best practices in health information security, including end-to-end data encryption, access control mechanisms, audit trails, and data anonymization techniques [26,27]. Strict adherence to HIPAA and privacy laws and careful attention to cyber security risks are essential to maintain patient and provider trust and legal compliance, as consultations may occur in non-secure environments [28].

Finally, clinicians in our study highlighted how administrative and legislative buy-in were necessary for real-world implementation, a finding supported by pediatric disaster groups attempting statewide implementation of specialty telehealth services [29]. Establishing trust and acceptance of disaster teleconsultation systems by the medical community and the public will require socialization and integration of the RDHRS program within the national strategic health response.

### Limitations

This study has limitations. First, the small, regionally restricted sample may limit generalizability. Still, we captured important perspectives from community and tertiary care providers in real-world conditions to guide future system refinements. Second, lack of opportunity to provide clinical care limited any exploration of how disaster teleconsultation services could impact patient outcomes, so more research is needed. Third, we focused on pilot implementation in Massachusetts, as this was the initial location of clinical need, so our analysis of barriers and facilitators may not apply to states or regions where laws or regulations differ. For example, states that allow license reciprocity or participate in regional compacts could streamline access to disaster teleconsultation services. This could benefit health professional shortage areas and rural communities where disasters are likely to exacerbate underlying disparities in healthcare access. To support future pilots in different state contexts, RDHRS and other regional disaster programs should establish processes to authorize disaster telehealth service delivery in coordination with local and state regulatory bodies and relevant government agencies. Last, hospital and clinician willingness to use regional disaster teleconsultation services for pandemics may differ from no-notice events where care delivery is time-sensitive and specialist supply is even more limited (eg, burn surgeons).

### Conclusions

Implementing RDHRS disaster teleconsultation services to connect field clinicians to specialists across state lines and hospital systems was feasible in real-world conditions. We improved system efficiency using a simple, device-agnostic, web-based platform to create a virtual MEOC that automatically routes, triages, and notifies experts of requested consultations. While user attitudes were generally positive, further technology and system refinements are needed to align call performance, reliability, and usability to desired standards. Although emergency waivers enabled the RDHRS to mobilize and deploy a virtual volunteer workforce for short-term disaster response, liability concerns and variable hospital credentialing practices delayed implementation. We encourage government and emergency management agencies to advance policies that expand liability protections for volunteer teleconsultants and incentivize hospitals to use standard procedures to support timely, effective regional disaster telehealth solutions.

## Abbreviations

HIPAA: Health Insurance Portability and Accountability Act
MEOC: medical emergency operations center
RDHRS: Regional Disaster Health Response System
REDCap: Research Electronic Data Capture
SME: subject matter expert
STROBE: Strengthening the Reporting of Observational Studies in Epidemiology
TUQ: Telehealth Usability Questionnaire
TUS: total usability score
